# Three-Month Mortality in Nonhaematological Patients with Chronic Pulmonary Aspergillosis: Differences between Subtypes

**DOI:** 10.3390/jof10100706

**Published:** 2024-10-10

**Authors:** Pablo González García, Julia Fernández-Navarro, Mónica Bru-Arca, Elisa Álvarez-Artero, Pablo Solís, María Pía Roiz Mesones, Juan Luis Muñoz Bellido, María Antonia García Castro, Moncef Belhassen-García, Javier Pardo Lledías

**Affiliations:** 1 Servicio de Medicina Interna, Hospital Universitario Marqués de Valdecilla, Instituto de Investigación Valdecilla (IDIVAL), Universidad de Cantabria, 39008 Santander, Spain; pablo.gonzalezg@scsalud.es; 2 Complejo Asistencial de Salamanca (CAUSA), Instituto de Investigación Biomédica de Salamanca (IBSAL), Universidad de Salamanca, 37007 Salamanca, Spain; juliafdeznavarro@gmail.com; 3 Servicio de Medicina Interna, Hospital Rio Carrión, 34005 Palencia, Spain; mbru@saludcastillayleon.es; 4 Servicio de Medicina Interna, Unidad de Enfermedades Infecciosas, Hospital Rio Carrión, 34005 Palencia, Spain; e.alvarez.artero@gmail.com; 5 Servicio de Medicina Interna, Hospital Universitario Marqués de Valdecilla, 39501 Santander, Spain; pablo.solis@scsalud.es; 6 Servicio de Microbiología, Hospital Universitario Marqués de Valdecilla, CIBERINFEC, ISCIII, IDIVAL, 39501 Santander, Spain; mpia.roiz@scsalud.es; 7 Servicio de Microbiología, CAUSA, IBSAL, Centro de Investigación de Enfermedades Tropicales de la Universidad de Salamanca (CIETUS), Universidad de Salamanca, 37007 Salamanca, Spain; jlmubel@usal.es; 8 Servicio de Microbiología, Hospital Rio Carrión, 34005 Palencia, Spain; mgarciacas@saludcastillayleon.es; 9 Servicio de Medicina Interna, Unidad de Infecciosas, CAUSA, IBSAL, CIETUS, Universidad de Salamanca, 37007 Salamanca, Spain

**Keywords:** aspergillus, pulmonary aspergillosis, chronic pulmonary aspergillosis, chronic cavitary pulmonary aspergillosis, chronic fibrosing pulmonary aspergillosis, chronic necrotising pulmonary aspergillosis

## Abstract

**Objectives**: Chronic pulmonary aspergillosis (CPA) is a fungal lung infection characterised by the slowly progressing destruction of the lung parenchyma and has four main subtypes. The objective of this work was to evaluate the epidemiology of CPA in our area and evaluate the involvement of the different subtypes in mortality. **Methods**: This was a descriptive longitudinal retrospective study developed in three tertiary hospitals in Spain. Among all patients admitted with a pulmonary aspergillosis diagnosis, we selected those who fulfilled the criteria for chronic aspergillosis according to the criteria of Denning, excluding those with a haematological disorder. **Results**: Among 409 inpatients recorded as having a pulmonary aspergillosis infection, only 76 (18.5%) fulfilled the criteria for CPA, with an estimated incidence of 0.67 cases/100,000 inhabitants/year. The subtypes detected were subacute invasive aspergillosis (SAIA) in 33 (43.4%) patients, simple aspergilloma (SA) in 25 (32.9%) patients, cavitary chronic aspergillosis (CCPA) in 13 (17.1%) patients, and chronic fibrosis (CFPA) in five (6.5%) patients. The overall three-month mortality rate was 23%, which was higher in SAIA patients. The predictors of early mortality were age > 65 years (OR 3.0 CI 95 1.0–9.5 *p* = 0.043) and the SAIA subtype vs. other subtypes (OR 3.1 CI 95 1.0–9.5 *p* = 0.042). **Conclusions**: The incidence rate estimated was inferior to that previously reported. The three-month mortality in patients with CPA was high, with older age and the SAIA subtype being the variable independent predictors of a worse prognosis.

## 1. Introduction

Pulmonary aspergillosis is the most common fungal lung disease. Its clinical presentation depends on host factors and the immune response. It ranges from milder forms, such as allergic bronchopulmonary aspergillosis (ABPA), to more severe and lethal forms, such as invasive pulmonary aspergillosis (IPA). An intermediate form is chronic pulmonary aspergillosis (CPA). It is characterised by the slowly progressing destruction of the lung parenchyma in patients with a history of structural lung disease, often with residual cavities, bullae, or scarring. The primary driver of disease in patients with CPA is the local immune response to the *Aspergillus* colonisation of previously damaged lung tissue. However, the immune response determines the clinical presentation. There are different subtypes: chronic cavitary pulmonary aspergillosis (CCPA), chronic fibrosing pulmonary aspergillosis (CFPA), subacute invasive aspergillosis (SAIA), formally called chronic necrotising pulmonary aspergillosis (CNPA), simple aspergilloma (SA), and aspergillotic nodules. In practice, CPA’s clinical syndromes and their radiology often overlap, and patients can transition from one form of CPA to another as their disease evolves over time [1,2]. Diagnostic methods are usually based on clinical presentation, radiological changes, and microbiological evidence [3]. The number of CPA cases worldwide has been estimated at three million [4], although the prevalence varies substantially by geography, with estimates of <1 case per 100,000 persons in Western Europe and the United States to 43 cases per 100,000 persons in Sub-Saharan Africa [5]. On the other hand, if we look at the series published in the literature, with few cases, these data could be highly overestimated. The impact of this fungal infection on mortality is also not well established, with a wide range in the series, from 4% to more than 50% [6,7]. Worse prognostic factors have also been described. However, it is not well known whether the SAIA subtypes constitute a group with a relatively poor prognosis. Thus, the aim of this study was to evaluate the incidence of CAP, its risk factors, and its mortality.

## 2. Methods

### 2.1. Design, Patients, and Setting

The design was a descriptive longitudinal retrospective study developed in three tertiary hospitals of the Spanish System of Health: Hospital Marques de Valdecilla (HUMV) in Cantabria, Complejo Asistencial Universitario de Palencia (CAUPA) in Palencia, and Complejo Asistencial Universitario de Salamanca (CAUSA) in Salamanca, located in Northern and Western Spain. These three hospitals cover a total risk population above 800,000 inhabitants. We selected all patients admitted between 2009 and 2022 to the three hospitals with a diagnosis of aspergillosis according to the ICD-9-CM, code 117.3, in 2009–2015, and the ICD-10, code B44, in 2016–2022. We included patients in the study if they fulfilled the criteria for chronic aspergillosis according to the criteria of Denning et al. [3]. Thus, we considered a simple aspergilloma (SA) if there was a single pulmonary cavity with a ball of fungal and serological or microbiological evidence of *Aspergillus* infection. Chronic cavitary pulmonary aspergillosis (CCPA) was considered if there was one or more pulmonary cavities with or without aspergilloma over a minimum of 3 months of observation and if there was serological or microbiological evidence of *Aspergillus* infection. Chronic fibrosing pulmonary aspergillosis (CFPA) was defined if the severe fibrotic destruction of two or more lobes of the lung was associated with CCPA. Subacute invasive aspergillosis (SAIA) was defined as the presence of cavitation or nodules and progressive consolidation occurring within 1–3 months of biopsy, with tissue invasion or positive *Aspergillus* galactomannan antigens in the blood or respiratory fluid. We excluded patients with haematological disorders if they had a definitive diagnosis of aplasia medullary or a haematologic malignancy or if they had undergone a bone marrow transplant.

### 2.2. Statistical Analysis

The results are expressed as percentages for categorical variables and as medians and interquartile ranges (IQRs) for continuous variables. The incidence rate of CPA was calculated in the defined time period of 2009–2022 by dividing the number of new cases of CPA by the total number of disease-free periods per 100,000 person-years. The denominators were obtained from the National Statistical Institute (INE in Spanish) (http://www.ine.es/, accessed on 2 May 2024). The chi-square test was used to assess associations among the categorical variables, such as mortality and other demographic variables, and the intensity of these associations was expressed as the odds ratio (OR) and CI 95% for the OR. Additionally, we applied the corresponding regression models for multivariate analysis. Patient survival was estimated via a Kaplan–Meier curve. Moreover, the log-rank test and the Cox proportional hazards model were used to assess differences between groups. We considered a statistically significant difference if the *p* value was <0.05. All of the data were analysed with SPSS 25 (Statistical Package for the Social Sciences).

## 3. Results

**Figure 1:**
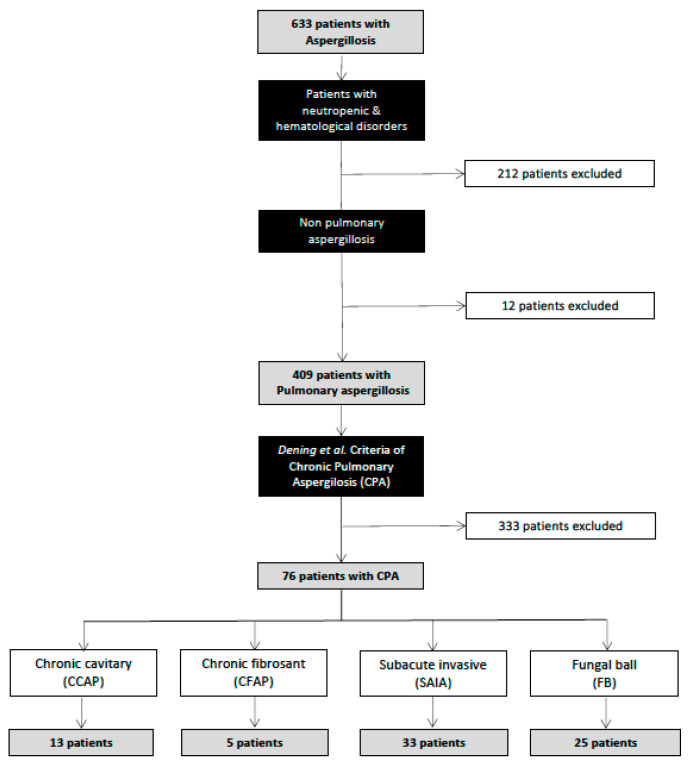
Flow chart of patients included in the study [3].

During the study period, 633 inpatients were recorded as having an aspergillosis infection at the three study hospitals (HUMV, CAUPA, and CAUSA). A total of 212 (33.5%) patients were excluded because of haematologic disorders, and 12 (1.8%) patients had aspergillosis in locations other than the lungs. Among 409 patients recorded as having pulmonary aspergillosis, only 76 (18.6%) fulfilled the criteria for a diagnosis of chronic pulmonary aspergillosis (CPA). Figure 1 shows the flow chart of the patients included in the study. Thus, the overall estimated incidence of CPA in our study area was 0.67 cases/100,000 inhabitants/year.

The patients with CPA included in the study are shown in Table 1.

The median age of the patients studied was 62 (IQR 60–66) years, and there were more men than women (male/female ratio 2.6). The subtypes most frequently detected were subacute invasive aspergillosis (SAIA) in 33 (43.4%) patients, simple aspergilloma (SA) in 25 (32.9%) patients, cavitary chronic aspergillosis (CCPA) in 13 (17.1%) patients, and chronic fibrosing pulmonary aspergillosis (CFPA) in five (6.6%) patients. We assessed the comorbidities among the patients with CPA (Table 1): chronic obstructive pulmonary disease (COPD) in 24 (31.6%) patients, diabetes mellitus in 17 (24%) patients, and remitted or active disease in 17 (24%) patients. A total of 54 (71%) patients used corticosteroids: 29 (38.2%) were systemic and 29 (38.2%) patients inhaled. Other immune suppressors were present in 24 (31.6%) patients, including 14 with mycophenolate, 3 with hydroxychloroquine, and 2 with methotrexate and anti-TNF alfa, among others. The symptoms and signs most frequently presented were dyspnoea (42, 55.3%), fever (22, 28.9%), and haemoptysis (18, 23.7%). The clinical and comorbidity differences between SAIA and other subtypes of CPA are shown in Table 2.

Previous tuberculosis was most common in the CCPA, CFPA, and SA groups, whereas the use of glucocorticoids and immunosuppressors and solid organ transplants were most common among patients with SAIA (*p* < 0.05).

**Figure 2:**
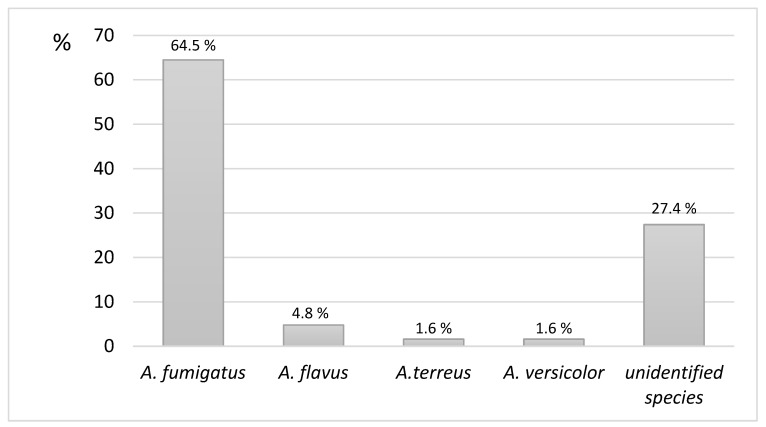
*Aspergillus* spp. involved in the cohort of chronic pulmonary aspergillosis.

The main microbiological species involved were *A. fumigatus* in 40 (64.5%) patients, *A. flavus* in 3 (4.8%) patients, and *A. terreus* and *A. versicolor* in 1 (1.6%) patient, whereas unidentified species were detected in 17 (27.4%) patients (Figure 2). Microbiological diagnoses in these cases were performed via histopathological methods in 38 patients (50%), whereas cultures of respiratory samples were available in 62 patients.

Antifungal therapy was used in 62 (81.6%) patients: voriconazole in 46 (60.5%) patients, itraconazole in 10 (15.3%) patients, and isavuconazole in 8 (10.5%) patients. Five patients had subtype SAIA and three had CCPA. Thirteen (17.1%) patients were treated with two or more antifungal agents. We did not find statistically significant differences in antifungal therapy between the different subtypes of CPA (*p* > 0.05).

**Figure 3:**
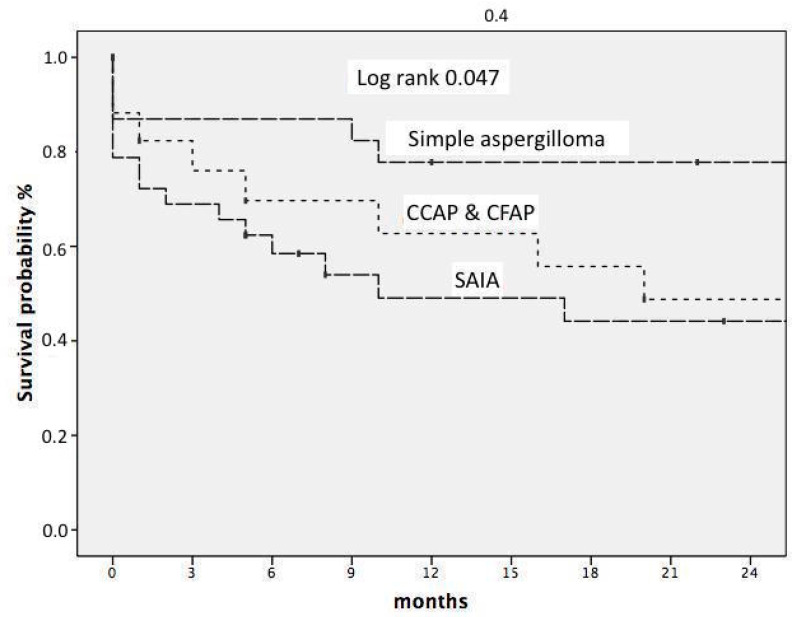
Survival probability at 24 moths.

We studied early mortality. Thus, the overall three-month mortality rate among patients with CPA was 23%, which differed among the three main subtypes, namely SA, CCPA and CFPA, and SAIA, with mortality rates of 12%, 16.6%, and 31.2%, respectively. The predictors of early mortality were age > 65 years (OR 3.0 CI 95 1.0–9.5 *p* = 0.043) and the SAIA subtype vs. other subtypes (OR 3.1 CI 95 1.0–9.5 *p* = 0.042). We also studied survival in this cohort, as shown Figure 3, with a mean follow-up period of 73 (RIQ 41–122) months. We found that age > 65 years (HR 4.1 (CI 95 1.9–8.6), *p* < 0.001), diabetes mellitus (HR 3.3 (CI 95 1.5–7.3) *p* = 0.003), the subtype of CPA SAIA vs. simple SA (HR 7.4 (CI 95 2.5–21.6), *p* < 0.05), and subtype CCPA/CFPC vs. SA (HR 4.9 (CI 95 1.9–12.5), *p* < 0.05) were independent predictors of a worse prognosis.

## 4. Discussion

In the last two decades, an increase in the incidence of aspergillosis has been reported, which is based on the prevalence of classical risk factors for invasive aspergillosis, as in the studies of admitted patients [8,9]. Different factors are involved. On the one hand, the use of new immune-suppressive therapies, such as biological drugs, small-molecule kinase inhibitors, or CAR-T-cell therapy, and epidemics caused by the influenza A-09 and SARS-CoV-2 viruses could be involved in increasing the incidence of invasive aspergillosis [10]. On the other hand, demographic factors, such as population ageing, could also contribute to the increase in the number of elderly people with different comorbidities, such as diabetes, COPD, or chronic kidney disease [9]. Unlike invasive aspergillosis, the classic factors associated with CPA, such as tuberculosis or pneumoconiosis, could be decreasing in Spain and other European countries [11,12]. Therefore, we currently do not know the epidemiological situation of CPA. In our work, we evaluated this in a retrospective study from 2009 to 2022 in three public hospitals. We selected all records of inpatients with a recorded diagnosis of pulmonary aspergillosis, excluding those with neutropenia or other haematological disorders. These patients usually undergo effective protocols for prophylaxis, early diagnosis, and pre-emptive treatment [13]. Thus, in our cohort, fewer than one-fifth of the patients with pulmonary aspergillosis met the CPA criteria, with an estimated incidence rate of less than 1 case/100,000 inhabitants/year. This incidence is lower than that reported by Maitre et al. [14], who used data from the database of French hospital discharge summaries and reported an incidence three times higher than that reported in our cohort. These differences could be explained by the methods used by these authors, because they accepted pulmonary patients with chronic aspergillosis recorded as code ICD-BB4. However, in this work, the authors did not evaluate the medical records of patients, evaluating whether the patients met the European CPA criteria, and assumed that all patients recorded met them. According to the data presented in our study and other papers [15], fewer than five inpatients classified as having aspergillosis fulfilled the criteria for CPA. Thus, we believe that the data presented by Maitre and colleagues could overestimate the current incidence, possibly due to classification bias. In our study, the median age of the patients included was 62 years, and the proportion was also higher in males than in females, which coincides with the majority of published articles [9,15,16]. A higher frequency of CCPA and simple aspergilloma than SAIA has been detected in the majority of studies in developing countries [17]. However, in high-rent countries, discordant results have been reported in the literature. Thus, some studies in Spain and other countries have reported a greater frequency of CCPA than SAIA [18,19]. In contrast, in two French studies with 44 and 24 patients, respectively, SAIA was the most frequent subtype, accounting for 50–62% of the total cases of CPA [14,20]. In our study, we classified the different subtypes according to the Denning criteria, and we also found a higher percentage of SAIA cases. The greater ratio of SAIA to other subtypes in our study area could be related to a shift in the epidemiological profile of patients with CPA, with a decrease in the incidence of tuberculosis and an increase in the number of patients receiving immunosuppressive therapy. This hypothesis is supported by our study and others [21], in which a strong association was found between the use of glucocorticoids or other immunosuppressants and SAIA, but there was no association with pulmonary tuberculosis. In contrast, other authors have not reported high exposure to glucocorticoids and other immunosuppressants in patients with SAIA. Hae-Seong Nam et al. reported, in a cohort of patients with SAIA, that only 19% received these treatments, and the main comorbidity detected was pulmonary tuberculosis, which was present in more than 90% of the patients [22]. Tuberculosis is considered the main comorbidity associated with CPA and affects 32 to 90% of patients [21,23]. In our work, we found that more than 20% of the patients underwent lung solid organ transplantation. Invasive aspergillosis and SAIA are considered complications in the first year after the procedure [24]. Differentiating between pulmonary aspergillosis invasion and SAIA is frequently difficult since both states of immunosuppression could be involved. However, a cavitary lung with subacute progression, which is usually associated with a halo, could be associated more frequently with the latter type [19]. An interesting finding in our study was the detection of CPA only in patients with pulmonary transplants, whereas we did not find cases in patients with other solid organ transplants, although lung transplants accounted for only 8% of the total solid organ transplants in Spain (www.ont.es, accessed 12 May 2024).

In terms of treatment, antifungals were administered to 62 patients (81.6%). The vast majority received voriconazole (74.2%), followed by itraconazole. Notably, the use of isavuconazole, a relatively recent broad-spectrum triazole antifungal, occurred in eight patients (12.9%), which is not an insignificant number compared with other studies where it is not even reflected. There are no published data on this, although it has been proposed as a third line of treatment [25]. These data open the possibility of further studies in this direction. Another aim of our study was to assess the overall mortality and its risk factors. Thus, we evaluated early mortality at 3 months and survival at 24 months. In a recent review, Lowes D. and colleagues reported significant differences in the mortality of CPA among different papers, ranging from 7% to 42% [7]. This fact could be due to methodological differences between the studies, according to the different types of patients included in each study and the follow-up time. In a large cohort of 387 patients in the UK, the survival rates were 86%, 62%, and 47% in 1, 5, and 10 years, respectively, with lower survival rates in patients with emphysema, a higher severity of breathlessness as determined by the Medical Research Council (MRC), resistance to azoles, and bilateral pulmonary effects [24]. In our study, we found higher early mortality rates than other studies did, with the mean age and the subtype of CPA being the only variables associated with mortality. Other works have also shown that older patients have a worse prognosis than younger patients [24]. Data in the literature concerning the prognosis according to the different subtypes of CPA are scarce. The mortality in patients with CCPA has generally been higher than that in patients with simple aspergilloma [24]. However, data concerning SAIA are lacking. In our work, patients with SAIA had higher 3-month mortality than did those in the other groups. Paradoxically, some papers have indicated that the SAIA subtype could have a better response to voriconazole than the CCPA subtype to other antifungals [26]. It is possible that the better radiological response is due to a greater fungal load and the absence of previous chronic lung lesions, such as fibrosis, cavities, or chronic pulmonary parenchymal destruction. However, given the invasive and necrotising nature of the SAIA subtype and the greater degree of immunosuppression in these patients [27], it is possible that SAIA patients have a better prognosis according to the clinical picture of invasive aspergillosis, because a worse final prognosis is not surprising.

In conclusion, despite the efforts of the European and American societies, there is currently still uncertainty in the diagnosis and classification of patients with CPA infection. Among the inpatients, in whom CPA was observed in over one-fifth of all patients, only one-fifth met the criteria for chronic pulmonary aspergillosis. The incidence rate estimated in our study is inferior to that previously reported. The three-month mortality in patients with CPA was high, with older age and the subacute invasive aspergillosis subtype being the variable independent predictors of a worse prognosis.

## Figures and Tables

**Table 1:** Main characteristics and comorbidities of patients with chronic pulmonary aspergillosis (CPA).

Variable	All Patients
Age median (RIQ), years	62 (IQR 60–66)
Male, n (%)	55 (72.4)
Comorbidities, n (%)	
Chronic obstructive pulmonary disease	24 (31.6)
Diabetes mellitus	17 (24)
Transplant solid organ	15 (19.7)
Interstitial lung disease	14 (18.4)
Bronchiectasis	10 (13.1)
Tuberculosis	12 (15.7)
Active neoplasm	6 (7.9)
Immunosupressors, n (%)	
Systemic glucocorticoids	29 (38.2)
Inhaled glucocorticoids	29 (38.2)
Other immunosuppressors	24 (31.6)
Symptoms, n (%)	
Dyspnoea	42 (55.3)
Fever	22 (28.9)
Hemoptysis	18 (23.7)
Subtype, n (%)	
Semi-invasive (SAIA)	33 (43.4)
Fungal ball (FBAP)	25 (32.9)
Cavitary chronic (CCAP)	13 (17.1)
Cavitary fibrosant (CFPA)	5 (6.6)
Nodule aspergilosis (NA)	0 (0)
Three-month mortality, n (%)	18 (23.7)

**Table 2:** Comparison of semi-invasive aspergillosis versus other forms of chronic pulmonary aspergillosis.

Variable Analysed	SAIA vs. Other CPA (%)	OR (CI 95)	*p*
Age > 64 years	42.4 vs. 44.2	0.9 (0.3–2.3)	0.878
Male, n (%)	66 vs. 76	0.7 (0.4–1.2)	0.33
Comorbidities, (%)			
Chronic obstructive pulmonary disease	27.3 vs. 34.9	0.8 (0.4–1.4)	0.479
Diabetes mellitus	24.2 vs. 20.9	0.8 (0.2–2.4)	0.731
Chronic kidney disease	12.1 vs. 4.7	1.6 (0.8–3.2)	0.23
Tuberculosis	0 vs. 27.9	ND	<0.001
Interstitial lung disease	24.2 vs. 11.6	2.4 (0.7–8.2)	0.148
Transplant solid organ	33.3 vs. 11.6	1.7 (1.1–2.8)	0.043
Immunosuppressors, (%)			
Systemic glucocorticoids	57.6 vs. 23.3	2.2 (1.3–3.6)	0.002
Inhaled glucocorticoids	33.3 vs. 41.9	0.8 (0.5–1.5)	0.65
Other immunosuppressors	45.5 vs. 20.9	1.8 (1.1–2.9)	0.023
Symptoms, (%)			
Hemoptysis	12.1 vs. 32.6	0.2 (0.1–0.7)	0.032
Dyspnoea	72.7 vs. 41.9	2.1 (1.1–4.0)	0.007
Fever	42.4 vs. 18.6	1.8 (1.1–2.9)	0.023
Three-month mortality, (%)	34.8 vs. 26.1	1.2 (0.6–2.1)	0.522

## Data Availability

Data are available upon reasonable request.
